# The buffering effects of mindfulness and organizational support on the mental health of hospital pharmacists in high-workload environments

**DOI:** 10.1038/s41598-025-96354-3

**Published:** 2025-04-08

**Authors:** Shazia Rehman, Khalid Abdullah Alotaibi, Erum Rehman, Muhammad Nasir Khan, Md Anisur Rahman, Bushra Yaqoob

**Affiliations:** 1 Department of Psychiatry, National Clinical Research Center for Mental Disorders, and National Center for Mental Disorders, The Second Xiangya Hospital of Central South University, Changsha, 410011 Hunan China; 2 Mental Health Institute of Central South University, China National Technology Institute on Mental Disorders, Hunan Technology Institute of Psychiatry, Hunan Key Laboratory of Psychiatry and Mental Health, Hunan Medical Center for Mental Health, Changsha, 410011 Hunan China; 3 Department of Psychology, College of Education, Prince Sattam bin Abdulaziz University, 11942 Al-Kharj, Saudi Arabia; 4 Department of Mathematics, Nazarbayev University, Nur-Sultan, Kazakhstan; 5 Electrical Engineering Department, Government College University Lahore, Lahore, 54000 Pakistan; 6 Department of Accounting, Data Analytics, Economics and Finance, La Trobe University, Melbourne, Australia; 7 Department of Pharmacy, The Women University, Multan, Pakistan

**Keywords:** Mental well-being, Workload, Mindfulness, Perceived organizational support, Hospital pharmacists, Mediation analysis, Psychology, Health care, Health occupations

## Abstract

The present research aims to explore the impact of workload on the mental well-being of hospital pharmacists, focusing on the mediating roles of mindfulness and perceived organizational support (POS). Utilizing a cross-sectional design, data were collected from 411 hospital pharmacists in Punjab, Pakistan, during July–September 2023 via stratified sampling. Mental well-being was assessed using the Patient Health Questionnaire (PHQ-9), the workload was assessed by the NASA Task Load Index (NASA-TLX), mindfulness using the Five Facet Mindfulness Questionnaire—Short Form (FFMQ-SF), and POS using a modified version of the Survey of Perceived Organizational Support (SPOS). Confirmatory Factor Analysis (CFA) was performed to validate the measurement model, ensuring the accuracy and reliability of the constructs involved. Further, the study utilized the PROCESS macro within SPSS (v28) to conduct mediation analysis, evaluating both direct and indirect effects of workload on mental well-being through the proposed mediators. The findings revealed a significant negative influence of workload on the participants’ mental well-being (β = − 0.472, *p* < 0.001). Three indirect pathways further affect this association: through mindfulness (β = − 0.208, *p* < 0.001), POS (β = − 0.135, *p* < 0.001), and serially through both mindfulness and POS (β = − 0.073, *p* < 0.001). These indirect effects collectively account for a substantial portion of the overall impact. The findings highlight the importance of implementing strategies to manage workload and enhance mindfulness and organizational support to promote the mental well-being of hospital pharmacists.

## Introduction

The healthcare sector is well-known for its high-stress environment, where professionals frequently encounter intense workloads^1^. In this context, hospital pharmacists play a crucial role in ensuring the safe and effective use of medications. However, their demanding workload can negatively impact their mental well-being and job performance^2,3^. This issue is a global concern, not confined to any particular region. Specifically, Punjab, a rapidly growing province in Pakistan’s healthcare sector, provides an ideal setting to explore these challenges comprehensively^4^. The mental well-being of hospital pharmacists is a vital component of their overall health and significantly influences their ability to deliver quality patient care^5^. Mental well-being includes emotional stability, resilience, and overall life satisfaction, and it is affected by various factors such as workload, workplace culture, and individual characteristics like mindfulness and perceived organizational support (POS)^6^.

The workload can be categorized into two primary dimensions: physical and mental characteristics^7^. However, assessing the relationship between these dimensions and work performance poses considerable challenges. An increase in workload, either in terms of intensity or duration of exposure, is associated with a heightened risk of illness or disease^8^. In examining dose–response relationships about workload, it is anticipated that the associations between workload and complaints will exhibit a U-shaped pattern^9^. Workload, particularly in the healthcare sector, is a significant stressor that can lead to burnout and reduced job satisfaction^10^. For hospital pharmacists, a high workload means managing complex medication regimens, dealing with prescription errors, and handling urgent requests, all while ensuring patient safety and compliance with regulations^11^. The COVID-19 pandemic further intensified these pressures by increasing demand for medication management, safety protocols, and vaccination support, which escalated both physical and psychological stress in healthcare settings^12^. Chronic workload stress is also known to disrupt restorative sleep cycles, which further exacerbates anxiety and depression symptoms by impairing emotional regulation and cognitive functioning. A neurophysiological study has demonstrated how stress impacts pathways regulating sleep behavior, linking sleep disruptions to mental health decline^13^. The relationship between workload and mental well-being among hospital pharmacists is complex and multifaceted. Prior evidence has suggested that workload is not only a source of stress but also a significant predictor of mental well-being outcomes^14–16^. However, the mechanisms through which workload affects mental well-being remain largely unexplored. This study aims to bridge this gap by examining the mediating roles of mindfulness and POS in this relationship.

Mindfulness, defined as the practice of non-judgmental present-moment awareness, has gained recognition as a potential buffer against the negative impact of workload on mental well-being^17,18^. Mindfulness-based programs, including Mindfulness-Based Stress Reduction (MBSR), necessitate intentionally directing their attention toward internal experiences, such as breath and bodily sensations, and external stimuli, including sounds and tastes, occurring in the present moment^19,20^. Breathing meditation exemplifies a traditional form of attention-based mindfulness practice that entails directing one’s attentional focus toward the breath. This practice stabilizes cognitive processes and enhances awareness of the present moment. A widely recognized form of attention-based meditation focuses on auditory stimuli, wherein the primary object of attention consists of the diverse sounds present within the surrounding environment. Mindfulness-based interventions have demonstrated efficacy in treating various mental disorders^21^. A variety of interventions have been established that demonstrate effectiveness in mitigating stress and chronic pain^22^, alleviating symptoms of depression^23^, preventing relapses associated with addiction^24^, and promoting healthier eating behaviors^25^. Although originally developed in clinical contexts, recent studies have explored how technology-based platforms can deliver mindfulness interventions, enabling broader access and benefits for those in high-stress environments^26^. Furthermore, evidence has suggested that they positively influence psychological resilience and enhance social cohesion factors, including interpersonal functioning^27,28^. The adaptation of mindfulness-based interventions to online platforms has demonstrated significant, albeit small to moderate, effects in mitigating symptoms of depression and anxiety, reducing stress, and enhancing overall mental well-being^29^.

Concurrently, compassion-based programs such as Mindful Self-Compassion (MSC) and Compassion-Focused Therapy (CFT) have been designed to enhance socio-affective competencies, including self-compassion, compassion for others, loving-kindness, prosocial motivation, altruism, and empathy. These programs strengthen personal and social resilience^30^. Compassion-based programs represent a synthesis of mindfulness-based exercises and compassion meditation practices. These programs typically exhibit an integrative and structured multi-component design explicitly aimed at fostering qualities such as acceptance, care, gratitude, and compassion^30,31^. The MSC program incorporates a variety of exercises designed to cultivate a compassionate inner dialogue, navigate challenging emotional experiences and interpersonal relationships, and align one’s actions with fundamental values. Compassion-based programs frequently integrate structured meditation practices, such as loving-kindness meditation, with informal interpersonal exercises, which encompass the sharing of self-critical and affectionate expressions among participants. Mindfulness interventions often target heightened awareness of sensory experiences to help reduce mental stress. This focus on bodily and sensory awareness aligns with studies that emphasize the importance of sensory experiences in shaping stress responses and well-being in other contexts. For instance, Qiao et al.^32^ have highlighted how multi-sensory experiences influence well-being in wheelchair users, suggesting that sensory factors play a critical role in stress management across various environments. Consequently. by promoting a state of calm and focused attention, mindfulness may enable individuals to manage stress more effectively and preserve their mental health despite heavy workloads.

Perceived organizational support (POS) is the degree to which employees believe their organization values their contributions and prioritizes their well-being, as evidenced by providing assistance and support^33^. The Organizational Support Theory (OST) posits that engagement within an organization constitutes a form of social exchange. This exchange dynamic encourages employees to contribute to the attainment of organizational objectives and to augment their work efforts in anticipation of receiving rewards^34^. As perceived by employees, organizational support constitutes a universal phenomenon that transcends any particular domain of activity within an organization. Chen et al.^3^ demonstrated that in the pharmaceutical industry, organizational support enhances employee engagement and mitigates stress, supporting improved mental well-being and performance outcomes. Employees who perceive support from the organization experience a sense of respect, characterized by recognizing their individual needs and limitations. This perception includes an acknowledgment of the effort that the employee dedicates to their work and the broader organizational environment. Furthermore, the organization’s willingness to forgive mistakes or temporary reductions in productivity—such as those resulting from illness—contributes to this supportive atmosphere. The organizational support theory is rooted in Blau’s social exchange theory. It is supported by empirical findings demonstrating a positive correlation between employees’ exertion of work effort and their perceptions of the reliability and equity of rewards^35^. After the publication of the Survey of Perceived Organizational Support (SPOS)^33^, a plethora of studies conducted globally have elucidated the importance of this construct and its interrelationship with various work-related and extraneous factors^36–38^. Occupational research indicates that employees who experience organizational support demonstrate enhanced performance^39^, exhibit greater job satisfaction^40^, display increased commitment and attachment to their roles, and are at a reduced risk of experiencing burnout^41^.

The concept of POS is intricately linked to employees’ emotional states and overall well-being. Studies have indicated substantial correlations between POS and various health-related outcomes, including general health status^42^, the frequency of somatic symptom complaints^43^, levels of anger^44^, stress, and self-esteem^45^. A study by Cao et al.^46^ also highlights how supportive environments can act as a buffer against early-life adversities, improving long-term mental resilience, a concept applicable to organizational settings as well. Furthermore, POS acts as an emotional buffer against the negative emotions associated with role conflict^47^. POS has been demonstrated to correlate with factors extending beyond the workplace, including employees’ psychological well-being^48^, overall life satisfaction^49^, and the ability to achieve a harmonious balance between work and familial responsibilities^50^. Likewise, POS has been recognized as a pivotal element in alleviating the negative impacts of workload. Hospital pharmacists who perceive strong organizational support may exhibit a greater capacity to manage elevated workloads while maintaining their mental well-being. Organizational support can manifest in multiple dimensions, including but not limited to recognition of employee contributions, provision of job security, and facilitation of opportunities for professional development.

The relatively high and growing healthcare demand in the rapidly expanding Punjab, Pakistan health sector might make hospital pharmacists more susceptible to challenges related to a heavy workload. Cultural, social, and economic factors specific to the province might also shape how well these professionals do their mental health. For example, cultural norms around work ethic and family responsibilities contribute to the burden that already falls on hospital pharmacists. This research intends to add insight in this direction by exploring the mediating roles of mindfulness and POS on the relationship between workload and mental well-being among hospital pharmacists in Punjab, Pakistan. The results from this study might be used by hospital administrators aiming to gift their pharmacy staff a safer work environment and increased mental health status that could ultimately improve patient outcomes as a positive workplace culture.

### Theoretical background

**Fig. 1:**
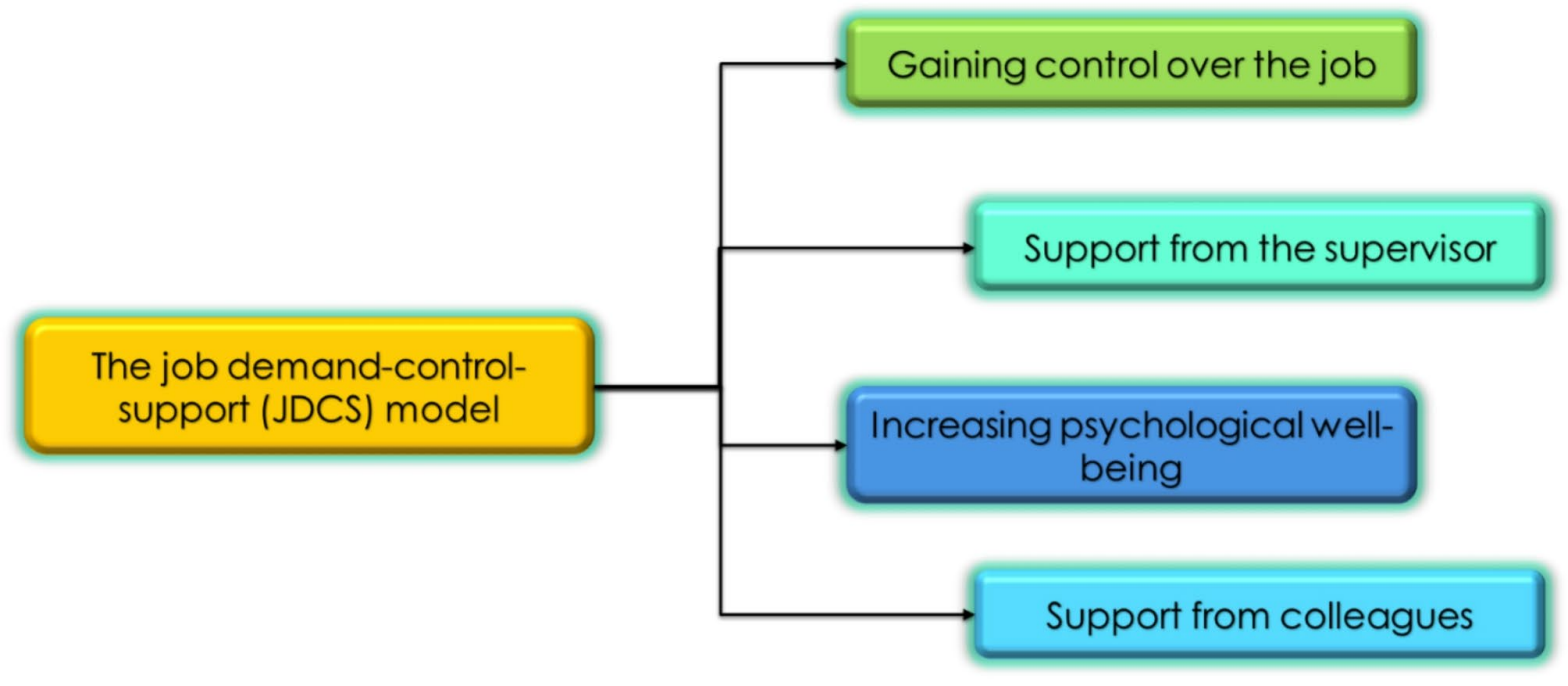
The job demand-control-support model. Description: The figure shows the job demand-control-support model dimensions^51,55,56^.

The Job Demand-Control-Support (JDCS) model proposes that job demands, such as workload, can lead to strain and diminished mental well-being, especially when coupled with low job control and inadequate support^51^. In high-stress professions like hospital pharmacy, the workload is particularly salient as it involves managing complex medication regimens, ensuring prescription accuracy, and addressing urgent requests, all of which contribute to elevated stress and burnout. Previous studies indicate that excessive workload negatively impacts mental well-being by depleting cognitive and emotional resources, leading to exhaustion and reduced psychological resilience^52,53^. Furthermore, pharmacists often experience limited recovery time between shifts, exacerbating the effects of workload and increasing the likelihood of long-term psychological distress^54^ Fig. 1.

#### Hypothesis 1

There is a negative effect of workload on the mental well-being of hospital pharmacists.

The elevated state of awareness, mindfulness, is attained through an intentional and non-judgmental focus on the present moment^57^. Exercise that incorporates kinesthetic awareness and physical exertion is called mindful exercise^58^. Individuals who engage in mindfulness practices demonstrate an enhanced capacity to regulate adverse emotions and intrusive thoughts. Furthermore, they exhibit an increased proficiency in assimilating and deriving insights from their experiences without evaluative judgment^59,60^. Numerous systematic reviews and meta-analyses suggested that interventions, such as mindfulness training, may enhance mental well-being. Furthermore, research indicates that a heightened level of mindfulness is significantly correlated with reductions in depression and stress^61–63^. This relationship improves self-regulation and a more optimistic perspective^64,65^. Mindfulness-based training has been the subject of extensive research regarding its efficacy in enhancing mental health and overall well-being. Mindfulness practices facilitate positive adaptation to adversity, thereby mitigating psychological distress, enhancing well-being, and improving mental well-being^66^. This study conceptualizes mindfulness as a mediator, helping pharmacists manage their workload by altering their perceptions and reactions to stressors. Additionally, strong organizational support may create an environment conducive to mindfulness practices, further enhancing their effectiveness in improving mental well-being.

#### Hypothesis 2 (H2)

Mindfulness positively mediates the relationship between workload and mental well-being of hospital pharmacists.

As defined by Eisenberger et al.^33^, POS refers to employees’ beliefs about their organization’s commitment to their well-being and value to the organization. According to the reciprocity norm in social exchange theory, employees who perceive higher organizational support are likelier to engage in positive workplace behaviors and experience better outcomes, including enhanced mental well-being. For hospital pharmacists, POS might mitigate the adverse effects of a high workload by providing a supportive environment that helps them cope with stress and feel valued, thereby preserving their mental wellness. While this study conceptualizes POS as a mediating variable grounded in its ability to enhance coping mechanisms and resilience, we acknowledge that POS could also serve as a moderator. As a moderator, POS might buffer the relationship between workload and mental well-being by weakening the direct adverse effects of workload. Future research could explore this alternative perspective to provide a more nuanced understanding of the role of POS in workplace dynamics.

As defined by Eisenberger et al.^33^, POS refers to employees’ beliefs about their organization’s commitment to their well-being and value to the organization. The subjective nature of POS has been extensively documented in research. Rhoades and Eisenberger^67^ argued that POS operates at the individual level because it is rooted in social exchange theory. According to this theory, when employees perceive strong organizational support, they feel an obligation to reciprocate through positive behaviors, such as increased effort and loyalty^68^. Conversely, employees who perceive low support may experience heightened stress, reduced motivation, and poorer mental health. This individual perception of support can influence psychological processes, including emotional regulation, resilience, and coping strategies, which mediate the relationship between job demands (workload) and mental well-being. Additionally, Kurtessis et al.^69^, in their meta-analysis of POS, emphasized that employees interpret organizational practices differently based on their unique psychological and situational context. These interpretations influence mental well-being by shaping how employees appraise and respond to stressors. When POS is high, employees may perceive stressful situations, such as high workload, as manageable due to the emotional and practical resources they believe are available to them. This can lead to improved resilience and reduced emotional exhaustion, thereby mitigating the negative effects of workload on mental well-being.

From a practical standpoint, studies have shown that POS enhances psychological safety by promoting a sense of security, belonging, and organizational fairness. These factors support psychological resources that help employees cope with job stress^70^. In this context, POS serves as a psychological mediator that connects organizational structures to individual mental health outcomes. For example, an employee who perceives strong support may benefit from greater self-efficacy and optimism in handling complex tasks, which buffers the impact of workload on stress. Thus, in this study, POS is hypothesized to function as a mediating variable rather than a moderator because it is measured based on individual perceptions and directly influences psychological mechanisms, such as stress appraisal, emotional regulation, and coping behavior^70^. While multilevel models can conceptualize POS as a shared organizational climate variable, this approach does not negate the established evidence of POS’s role as an individual-level factor. Consequently, consistent with prior research^67,69,70^, POS can mediate the relationship between workload and mental well-being, reflecting how employees internalize organizational policies and support structures within their psychological framework.

#### Hypothesis 3 (H3)

POS positively mediates the relationship between workload and mental well-being of hospital pharmacists.

The combined impact of individual and organizational resources, mindfulness, and POS provides a deeper understanding of how workload affects mental well-being. According to the JDCS model, the adverse effects of high workload can be mitigated by personal and organizational resources^51^. As a personal resource, mindfulness enhances emotional regulation and stress resilience^17^, while POS, as an organizational resource^33^, provides a sense of value and care, fostering psychological well-being. Integrating these two mediators creates a sequential pathway, offering a holistic perspective on mitigating workload-related stress.

By improving emotional regulation and stress resilience, mindfulness enhances pharmacists’ awareness and ability to perceive organizational support. Pharmacists with higher mindfulness are more likely to recognize and benefit from the support provided by their organization, such as access to resources, fair policies, and emotional backing. This enhanced perception of POS is an additional buffer against workload stress, ultimately protecting mental well-being. This sequential mediation mechanism highlights the interplay between personal and organizational factors, illustrating how they shape psychological outcomes in high-stress environments. Empirical evidence supports this combined pathway. For instance, Hulsheger et al.^71^ demonstrated that mindfulness interventions improved resilience and job satisfaction, indirectly reducing stress levels. Similarly, Rhoades and Eisenberger^67^ found that POS significantly alleviates strain when combined with personal coping strategies, creating a cascading effect on well-being. Shonin et al.^72^ further emphasized that mindfulness facilitates greater recognition and utilization of organizational support, amplifying its protective effects. These findings align with the broader theoretical frameworks of the JDCS model, mindfulness theory, and social exchange theory, underscoring the value of integrating personal and organizational resources to improve mental well-being. This leads to the fourth hypothesis:

#### Hypothesis 4

There is a chain mediation effect of mindfulness and POS between workload and mental well-being of hospital pharmacists.

#### Study gaps and contribution

Hospital pharmacists are at the forefront of ensuring patient safety and treatment efficacy, yet their mental health remains a significantly underexplored area of research, particularly in the context of developing regions like Punjab, Pakistan. This study focuses on workload, a pervasive and critical issue in the pharmacy profession, which encompasses long working hours, complex medication regimens, urgent patient demands, and error-prone environments. These stressors affect pharmacists’ professional performance and affect their mental well-being, making it imperative to understand their implications comprehensively.

Existing literature extensively documents the relationship between workload and mental health outcomes, but it primarily focuses on broader healthcare professions^73^, with pharmacists often receiving less attention. Additionally, while interventions like mindfulness and organizational support have been studied individually, there is limited understanding of how these factors interact to mediate the effects of workload on mental well-being. Specifically, few studies investigate the dual roles of mindfulness and POS in mitigating the adverse effects of workload on mental health. Furthermore, research addressing these dynamics within culturally distinct environments, such as Punjab, remains sparse despite the region’s unique socio-cultural landscape, which includes familial responsibilities, societal expectations, and systemic challenges in the healthcare system.

**Fig. 2:**
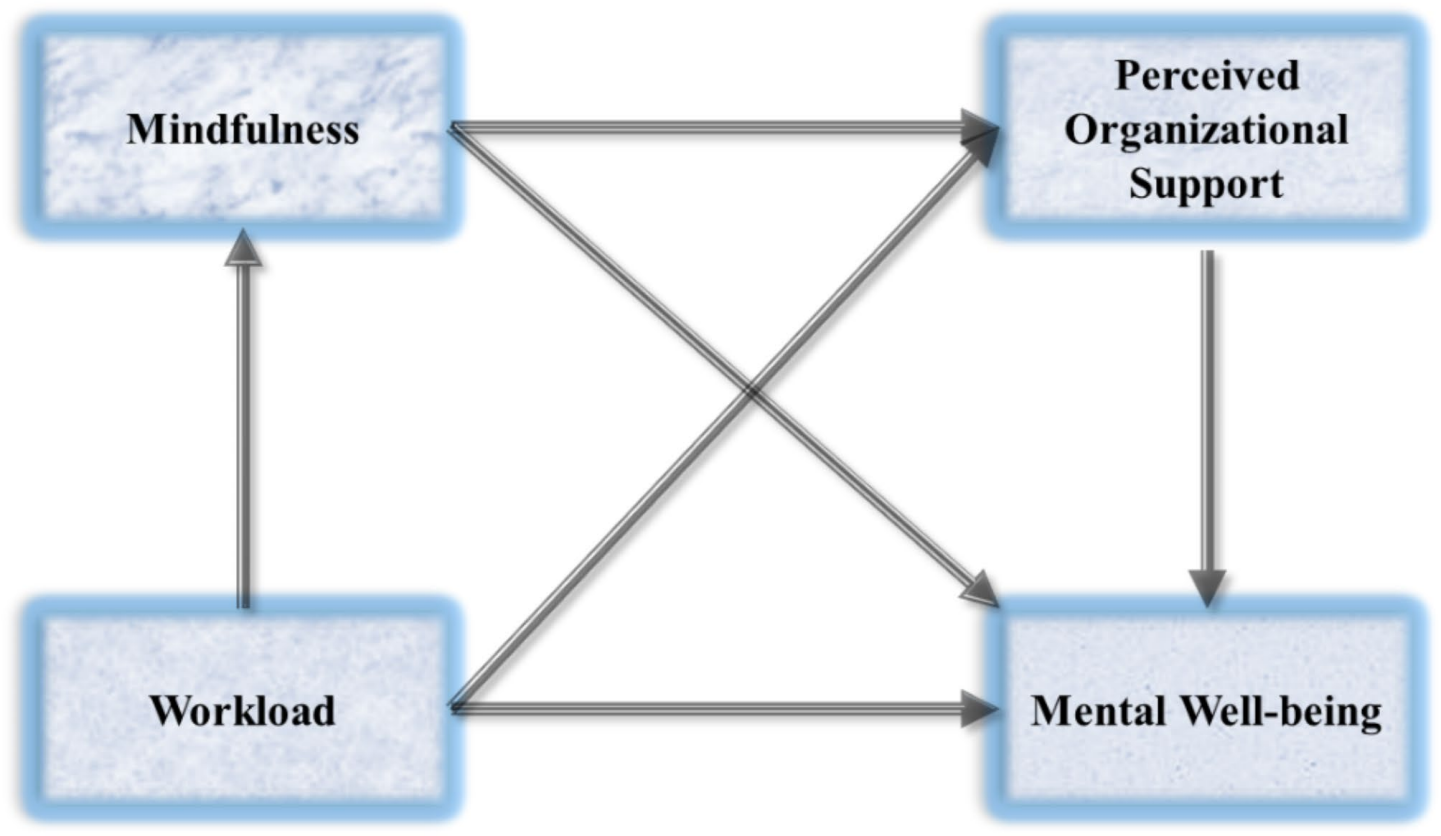
Hypothesized study model.

This study addresses these gaps by examining how mindfulness and POS mediate the relationship between workload and mental well-being among hospital pharmacists in Punjab, Pakistan (Fig. 2). Integrating the JDCS model, mindfulness theory, and social exchange theory, this research offers a holistic framework to understand how personal and organizational resources influence mental health outcomes. The focus on Punjab also provides a unique lens for understanding how cultural and contextual factors amplify or mitigate these relationships, offering insights that may not be generalizable from studies conducted in Western or urban contexts.

Additionally, this research seeks to contribute to the growing body of evidence on mental health interventions by providing actionable insights for healthcare policymakers and administrators. By identifying the fundamental mechanisms—mindfulness and POS—that support pharmacists’ mental well-being, the study underscores the need for tailored interventions, such as mindfulness training programs and workplace support systems. These findings have the potential to inform broader strategies for improving healthcare workforce resilience, particularly in resource-constrained settings. In summary, this study fills a critical gap in the literature by:

- Investigating the unique stressors faced by hospital pharmacists in Punjab and their impact on mental health.
- Exploring the combined mediating roles of mindfulness and POS in the workload-mental health relationship.
- Integrating theoretical models (JDCS, mindfulness, and social exchange theories) to provide a comprehensive framework for understanding these dynamics.
- Addressing the socio-cultural context of Punjab, which has received limited attention in mental health and workload research.
- Offering practical recommendations for healthcare organizations to foster mental well-being among pharmacists through targeted interventions.

## Methods

### Data source and study population

The data for this study were sourced from hospital pharmacists working in Punjab province, Pakistan, during July–September 2023. A comprehensive list of provincial hospitals was compiled using the provincial health directory and online databases. This list formed the basis for our sampling frame and included pharmacists from various hospital settings such as tertiary care hospitals, district headquarters hospitals, and rural healthcare centers.

### Sampling

A stratified random sampling technique was employed to ensure representation from diverse hospital settings and geographical areas within Punjab. Hospitals were stratified based on location (urban/rural) and type (tertiary care/district headquarters/rural healthcare center). Hospitals were randomly selected from each stratum using a computer-generated random number list. All pharmacists in the selected hospitals were invited to participate in the study.

### Sample size calculation

Utilizing Cochran’s formula for finite populations, the sample size was determined by considering the total number of pharmacists in Punjab province. Assuming a 95% confidence level, a margin of error of 5%, and a response distribution of 50%, the minimum required sample size was estimated to be 377. To offset potential non-responses, the goal was to enlist 411 pharmacists. This method ensured that the sample reflected the larger population of hospital pharmacists in Punjab province, thereby allowing for inferences about their experiences and viewpoints with a significant level of assurance.

### Eligibility criteria

Participants were included in the study if they were professionally qualified pharmacists with a valid license to practice in Pakistan and were currently employed in a hospital setting within Punjab province. This includes full-time, part-time, and contract workers with at least one year of professional experience in a hospital setting and directly involved in patient care or medication management. Participants were required to provide written informed consent to participate in the study. They must be willing to participate, understand the study aims and procedures, and their rights as participants, including the right to withdraw at any time without consequence.

Participants with diagnosed mental illnesses or those currently receiving treatment for a mental health condition were excluded from the study. This was necessary to avoid confounding factors affecting mental well-being, workload, mindfulness, POS, and stress levels. Furthermore, pharmacists who were on leave (e.g., maternity leave, sick leave) during the data collection period were also excluded to ensure the relevance and consistency of the data.

### Data collection

Primary data was collected through self-administered, structured questionnaires to assess diverse facets of pharmacists’ workload, mental well-being, mindfulness, and POS. To streamline the data collection process, the research team engaged with selected hospitals in person and via email, elucidating the study’s objectives and methodologies and procuring necessary authorizations. On-site recruitment and data collection were spearheaded by research assistants who visited the hospitals, briefed participants about the study, and secured written informed consent from the participating pharmacists. For participants recruited via email, a digital version of the information sheet and consent form were disseminated, and consent was obtained electronically.

**Fig. 3:**
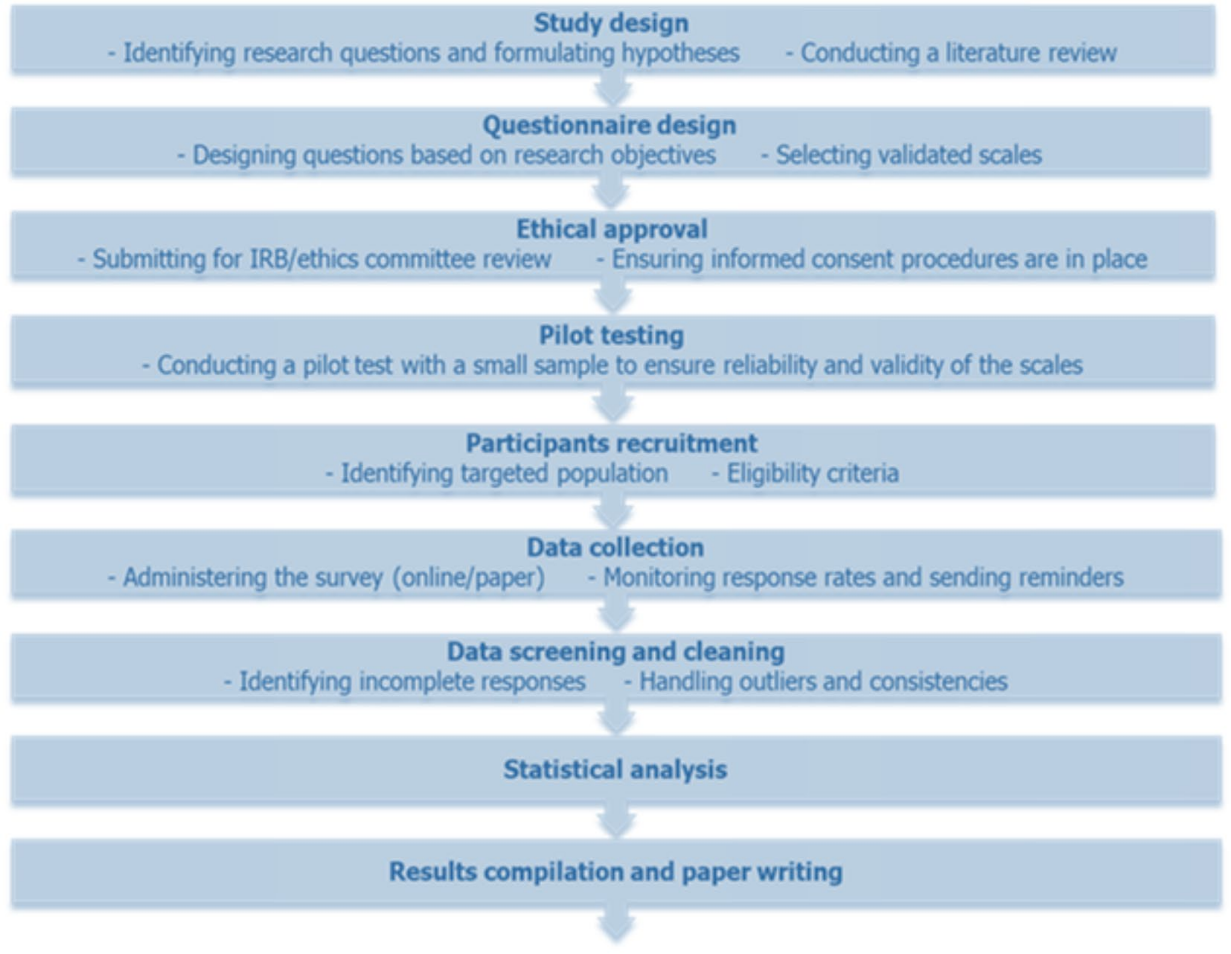
Study flowchart

Upon their discretion, participants were granted the autonomy to complete the questionnaire in a tangible format or digitally. For those electing the former, questionnaires were disseminated during personal encounters and reclaimed by research assistants. As for online engagement, a secure web link to the questionnaire was furnished, with hosting on a password-secured platform to safeguard data integrity and participant anonymity. In both scenarios, participants were guaranteed the confidential handling of their submissions and apprised that their involvement was voluntary. Furthermore, they were notified of their prerogative to retract from the study without explanation. The accumulation of completed questionnaires spanned four weeks, complemented by periodic follow-ups to optimize response frequency. Post-collection, all hardcopy questionnaires were digitized and incorporated into an electronic repository, wherein the data, encompassing both physical and digital submissions, were consolidated for subsequent analysis (Fig. 3).

### Data cleaning and handling of missing data

After the completion of data collection, responses were screened for errors, missing values, and outliers. Duplicate entries were removed, and all physical responses were cross-verified during data entry to ensure accuracy. Responses with more than 10% missing values in key variables were excluded from the analysis. For responses with minimal missing data, mean imputation was applied to fill in single missing values for non-critical variables (e.g., demographic items). Core variables related to mental well-being, workload, mindfulness, and POS were analyzed without imputation to preserve the validity of the results. This cleaning process ensured a consistent and high-quality dataset for statistical analysis.

### Ethical approval

The study was conducted in accordance with ethical standards, and approval was obtained from the Ethics Review Committee (ERC) of the Women University, Multan (ref#WU-09438/17). Participants were provided with detailed information about the study’s objectives, procedures, and rights as participants, including the option to withdraw at any point. Written informed consent was obtained from all participants prior to data collection. All data were anonymized, and strict confidentiality protocols were maintained throughout the research process.

### Measurement scales

#### Mental well-being (dependent variable)

The mental well-being of the study participants was assessed using the Patient Health Questionnaire (PHQ-9), which is a reliable and valid 9-item scale used for assessing the presence and severity of depression symptoms, with scores ranging from 0 to 27^74^. It aligns with DSM-IV criteria for Major Depressive Disorder and has a Cronbach’s alpha of 0.89, demonstrating high internal consistency^75–77^. Scores are categorized as no depression (0–4), mild (5–9), moderate (10–14), moderately severe (15–19), and severe depression (20–27). We utilized the PHQ-9 in our study to evaluate participants’ depressive symptoms effectively.

#### Mindfulness (mediator 1)

This study utilized the Five Facet Mindfulness Questionnaire—Short Form (FFMQ-SF) to measure mindfulness. The FFMQ-SF is a 24-item questionnaire that comprehensively assesses mindfulness by measuring five distinct facets: Observing, Describing, Acting with Awareness, Non-Judging, and Non-Reacting. These facets collectively capture the multidimensional nature of mindfulness as a construct^78^. The short form was developed by Bohlmeijer et al.^79^ and has been validated for use in various populations, demonstrating good psychometric properties^80–82^. Responses to the FFMQ-SF are collected using a 5-point Likert scale, ranging from 1 (“Never or Very Rarely True”) to 5 (“Very Frequently or Always True”). Participants are instructed to respond to each item based on their typical experiences over the last month. To obtain the final average score for each facet, the scores for the individual items within each facet are summed and then divided by the number of items in that facet. The facet scores can be interpreted independently or combined to obtain an overall mindfulness score. Higher scores on the FFMQ-SF indicate higher levels of mindfulness.

#### Perceived organizational support (mediator 2)

This study utilized a modified version of the Survey of Perceived Organizational Support (SPOS) consisting of 10 items to assess POS. The SPOS is designed to measure the extent to which employees believe their contributions are valued by their organization and that the organization cares about their well-being. The scale was developed by Eisenberger et al.^33^ and has been widely adopted in organizational behavior research, including the Pakistani population^6,83,84^, demonstrating robust psychometric properties. Responses to the SPOS are collected using a 7-point Likert scale, ranging from 1 (“Strongly Disagree”) to 7 (“Strongly Agree”). Participants are instructed to respond to each item based on their perceptions of their organization’s support. The scores for the individual items are summed to obtain the final score. A higher total score indicates higher levels of POS.

#### Workload

The NASA Task Load Index (NASA-TLX) is a widely recognized tool used to assess the workload experienced by individuals subjectively during task performance. Originally developed by the Human Performance Group at NASA’s Ames Research Center in the late 1970s and early 1980s, the NASA-TLX provides a systematic method for measuring perceived workload across six dimensions: mental demands, physical demands, temporal demands, own performance, effort, and frustration level^85^. These dimensions are combined into an overall workload score ranging from low to high, offering insights into a given task’s cognitive and physical requirements. To use the NASA-TLX, study participants typically complete a series of pairwise comparisons between the different workload dimensions, which helps to determine the weighting of each dimension in contributing to the overall workload. Participants rate their experience on each dimension using a visual analog scale with values ranging from 0 to 100 (0–9: Very Low, 10–29: Low, 30–49: Medium, 50–79: High, 80–100: High). The NASA-TLX has demonstrated good reliability and validity across different populations^86–88^.

### Statistical approach

Statistical analyses were performed using SPSS Version 28 and the PROCESS macro for mediation analysis. The Kaiser–Meyer–Olkin (KMO) measure of sampling adequacy was applied to ensure the data’s suitability for factor analysis, while Bartlett’s Test of Sphericity verified that the correlation matrix was appropriate for this analysis. Confirmatory factor analysis (CFA) was conducted to confirm that the measurement model adhered to established fit criteria. Descriptive statistics, including mean, standard deviation, skewness, and kurtosis, were calculated to assess the normality of the data distribution. Bivariate correlations were examined to explore the relationships between workload, mindfulness, perceived organizational support, and mental well-being. Multiple regression analysis was utilized to investigate the direct effects of workload on mental well-being and to assess the mediating effects of mindfulness and perceived organizational support. The model included age and gender as controlled variables. Mediation analysis using the PROCESS macro was performed to determine the indirect effects of workload on mental well-being through the proposed mediators. The bias-corrected bootstrap approach was applied to test the mediation hypothesis on a sample of 5000.

## Results

### Model adequacy analysis

The Kaiser–Meyer–Olkin (KMO) Measure of Sampling Adequacy has a value of 0.811, which is deemed very good for conducting factor analysis. Additionally, Bartlett’s Test of Sphericity yields a significant outcome (Sig. 0.000), suggesting that the correlation matrix deviates from an identity matrix, confirming the data’s suitability for factor analysis (Table 1).

**Table 1:** KMO and Bartlett’s test of sphericity.

KMO and Bartlett’s Test		
Kaiser–Meyer–Olkin Measure of Sampling Adequacy		0.811
Bartlett’s Test of Sphericity	Approx. Chi-Square	583.48
	df	410
	Sig	0.000

### Confirmatory factor analysis

Table 2 represents the summary of the constructs’ measurement properties. All four examined constructs—mental well-being, mindfulness, POS, and workload—display robust reliability and adequate convergent validity. This is evidenced by their respective Cronbach’s α, Composite Reliability (CR), and Average Variance Extracted (AVE) values. The standardized factor loadings for each item within these constructs signify that the individual items considerably contribute to their corresponding constructs. Significantly, most loadings exceed the 0.7 threshold, indicating high item reliability. Although one item within the mindfulness construct presents a slightly lower loading of 0.667, the overall reliability and validity of the construct remain significantly robust. These findings validate the measurement models for mental well-being, mindfulness, POS, and workload, confirming their suitability for further empirical investigation.

**Table 2:** Summary of construct measurement properties.

Construct	Standardized factor loadings	Cronbach’s α	CR***(> 0.7)	AVE***(> 0.5)
Mental well-being		0.91	0.943	0.636
MWB-1	0.734			
MWB-2	0.845			
MWB-3	0.762			
MWB-4	0.811			
MWB-5	0.789			
MWB-6	0.823			
MWB-7	0.753			
MWB-8	0.867			
MWB-9	0.782			
Mindfulness (MF)		0.89	0.972	0.592
MF-1	0.722			
MF-2	0.853			
MF-3	0.781			
MF-4	0.912			
MF-5	0.667			
MF-6	0.891			
MF-7	0.743			
MF-8	0.836			
MF-9	0.701			
MF-10	0.774			
MF-11	0.824			
MF-12	0.685			
MF-13	0.887			
MF-14	0.903			
MF-15	0.791			
MF-16	0.953			
MF-17	0.768			
MF-18	0.816			
MF-19	0.874			
MF-20	0.731			
MF-21	0.803			
MF-22	0.692			
MF-23	0.842			
MF-24	0.756			
Perceived organizational support (POS)		0.90	0.948	0.647
POS-1	0.756			
POS-2	0.812			
POS-3	0.879			
POS-4	0.742			
POS-5	0.831			
POS-6	0.763			
POS-7	0.850			
POS-8	0.729			
POS-9	0.865			
POS-10	0.798			
Workload (WL)		0.87	0.914	0.639
WL-1	0.726			
WL-2	0.841			
WL-3	0.768			
WL-4	0.857			
WL-5	0.784			
WL-6	0.813			

***p<0.001.

### Model fit analysis

Table 3 demonstrates the outcomes of model fit analysis of the study constructs. The mental well-being construct demonstrates an excellent fit, indicating minimal discrepancies between the observed data and the model. Mindfulness also shows a strong fit, while POS is satisfactory. Although the workload model is the least optimal, it remains acceptable. The overall model exhibits an excellent fit, highlighting a robust representation of the combined constructs.

**Table 3:** Model fit analysis.

	RMSEA	SRMR	CFI	TLI	${\chi }^{2}/df$
Mental well-being	0.035	0.045	0.992	0.985	1.29
Mindfulness	0.041	0.032	0.991	0.995	1.35
Perceived organizational support	0.043	0.052	0.972	0.983	1.26
Workload	0.053	0.061	0.952	0.971	1.54
Overall	0.025	0.035	0.997	0.993	1.32

### Descriptive analysis

The provided values in Table 4 suggest that all four constructs approximate a normal distribution. Mental well-being, with a mean of 13.56 and a standard deviation of 3.25, exhibits slight negative skewness (-0.5) and near-normal kurtosis (0.2). Mindfulness, which has a mean of 31.43 and a standard deviation of 2.87, shows slight positive skewness (0.3) and a flatter distribution (kurtosis of -1.0). POS, with a mean of 30.86 and a standard deviation of 6.23, displays mild positive skewness (0.4) and moderate kurtosis (0.6). Workload, characterized by a mean of 12.73 and a high standard deviation of 52.05, exhibits minimal skewness (0.1) and near-normal kurtosis (0.2). These statistics indicate that, despite some variability, these constructs’ distributions are generally normal.

**Table 4:** Descriptive analysis of study constructs.

	Mean	Standard deviation	Skewness (− 1, + 1)	Kurtosis (− 2, + 3)
Mental well-being	13.56	3.25	− 0.5	0.2
Mindfulness	31.43	2.87	0.3	− 1.0
Perceived organizational support	30.86	6.23	0.4	0.6
Workload	12.73	52.05	0.1	0.2

### Correlational analysis

Table 5 presents the bivariate correlation analysis of the study variables. The findings reveal a significant positive correlation between mental well-being and mindfulness (r = 0.61, *p* < 0.001) and POS (r = 0.57, *p* < 0.001), suggesting that individuals who engage in mindfulness practices and perceive more significant levels of organizational support are more likely to report higher levels of mental well-being. The findings indicate a moderate positive correlation between mindfulness and POS, with a correlation coefficient of 0.52 (*p* < 0.001). This suggests that individuals who exhibit a higher degree of mindfulness also tend to experience greater perceived support from their organizations. In contrast, the workload is moderately negatively correlated with mental well-being (r = -0.43, *p* < 0.001) and has weaker negative correlations with mindfulness (r = -0.33, *p* < 0.01) and POS (r = -0.28, *p* < 0.01). This suggested that higher workloads adversely affect employees’ mental well-being, mindfulness practices, and perceptions of organizational support.

**Table 5:** Pearson’s bivariate correlation analysis of the study construct.

	Mental well-being	Mindfulness	Perceived organizational support	Workload
Mental well-being	1			
Mindfulness	0.61***	1		
Perceived organizational support	0.57***	0.52***	1	
Workload	− 0.43***	− 0.33**	− 0.28**	1

***p<0.001, **p<0.01.

### Regression analysis

The regression analysis findings demonstrate statistically significant associations among age, gender, workload, mindfulness, POS, and mental well-being, as presented in Table 6. The results indicated a positive correlation between age and mindfulness (β = 0. 215, t = 2.37, *p* < 0.01), POS (β = 0.185, t = 1.76, *p* < 0.05), and mental well-being (β = 0.206, t = 2.25, *p* < 0.01). This suggests that older individuals are more likely to engage in mindfulness practices, perceive higher levels of support from their organization, and report greater mental well-being. Gender demonstrated a negative correlation with mindfulness (β = − 0.147, t = − 1.97, *p* = 0.304), POS (β = − 0.121, t = − 1.54, *p* < 0.183), and mental well-being (β = − 0. 133, t = − 1.75, *p* < 0.225), suggesting that females exhibit lower scores on these measures in comparison to males.

**Table 6:** Regression analysis results.

	Mindfulness		Perceived organizational support		Mental well-being	
	β	t	β	t	β	t
Age	0.215	2.37**	0.185	1.76*	0.206	2.25**
Gender	− 0.147	− 1.97	− 0.121	− 1.54	− 0.133	− 1.75
Workload	− 0.364	− 4.78***	− 0.311	− 4.37***	− 0.472	− 5.34***
Mindfulness			0.460	5.51***	0.571	6.41***
Perceived organizational support					0.434	5.21***
R^2^	0.34	0.44	0.49			
F	17.238***	26.784***	30.052***			

***p<0.001, **p<0.01, *p<0.05.

Further, the results indicate a negative correlation between workload and mindfulness (β = − 0.364, t = − 4.78, *p* < 0.001), POS (β = − 0.311, t = − 4.37, *p* < 0.001), and mental well-being (β = -0.472, t = -5.34, *p* < 0.001). These findings suggest that increased workloads adversely affect employees’ mindfulness, perceptions of organizational support, and mental well-being. This research findings indicate a positive correlation between mindfulness and POS (β = 0.46, t = 5.51, *p* < 0.001) and mental well-being (β = 0.571, t = 6.41, *p* < 0.001). It underscores the significance of mindfulness in improving perceptions of organizational support and fostering improved mental well-being. The study findings demonstrate a significant positive association between POS and mental well-being (β = 0.434, t = 5. 21, *p* < 0.001), highlighting the vital role of organizational support in promoting improved mental well-being among workers. The model demonstrates significant explanatory power, accounting for 34% of the variability in mindfulness, 44% in POS, and 49% in mental well-being.

### Serial mediation analysis

The findings from the mediation analysis indicate a multifaceted connection between workload and mental well-being, involving direct and indirect mediating pathways within a serial mediation framework (Table 7). The findings indicated that an augmented workload significantly impacts mental well-being, leading to a decrease in overall mental well-being levels, accounting for 53.09% of the total effect. Furthermore, the results suggest a significant serial mediation process in which workload impacts mental well-being by way of two consecutive mediators: mindfulness and POS. The serial mediation pathway contributes an additional 8.21% to the total effect. Increased workloads have been found to negatively impact an individual’s mindfulness, resulting in diminished perceptions of organizational support and ultimately leading to decreased mental well-being.

**Table 7:** The Mediation results.

	Effect size	Boot LCI	Boot ULCI	Ratio (%)
Direct path				
Workload → Mental well-being	− 0.472	− 0.55	− 0.39	53.09
Indirect paths				
Workload → Mindfulness → Mental well-being	− 0.208	− 0.26	− 0.15	23.40
Workload → Perceived organizational support → Mental well-being	− 0.135	− 0.16	− 0.11	15.19
Workload → Mindfulness → Perceived organizational support → Mental well-being	− 0.073	− 0.09	− 0.05	8.21
Total indirect effect	0.416	0.39	0.44	46.79
Total effect	0.889	0.77	0.932	100

Boot SE, Bootstrap standard error; Boot LCI, Bootstrap lower confidence interval; Boot UCI, Bootstrap upper confidence interval.

**Fig. 4:**
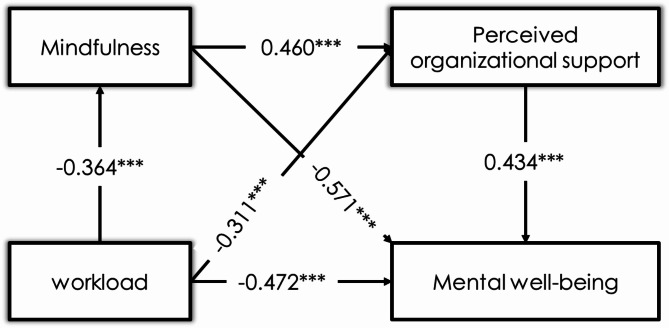
Path analysis of the study constructs.

Furthermore, the results show two simple mediation pathways: mindfulness and POS. These pathways account for 23.40% and 15.19% of the total effect, respectively. In these pathways, increased workload negatively impacts mental well-being by reducing either mindfulness or POS. Collectively, the direct effect and the three indirect pathways (through mindfulness, through POS, and both mindfulness and POS in sequence) explain 100% of the total effect of workload on mental well-being. These findings underscore the importance of considering both the direct and indirect effects of workload on mental well-being and highlight the role of mindfulness and POS as key mediating factors (Fig. 4).

## Discussion

The primary objective of this study was to investigate the impact of workload on the mental well-being of hospital pharmacists in Punjab, Pakistan, focusing on understanding the mediating roles of mindfulness and POS. By employing a serial mediation model, this study aimed to provide a nuanced understanding of how individual and organizational resources interact to mitigate the adverse effects of workload. This investigation is particularly critical in the unique socio-cultural and resource-constrained context of Punjab, where pharmacists face dual stressors of professional and familial obligations. The findings revealed a significant negative effect of workload on mental well-being, indicating that higher workloads are associated with decreased mental well-being. This association is further complicated by three indirect pathways: through mindfulness, through POS, and serially through both mindfulness and POS. These indirect effects collectively accounted for a substantial portion of the overall impact, underscoring the importance of considering both individual and organizational factors when assessing the mental well-being of pharmacists.

### The direct impact of workload on mental well-being leading to study hypothesis 1

The first hypothesis of the negative effect of workload on pharmacists’ mental well-being was consistent with prior scholarly investigations. According to an empirical investigation, pharmacists in Pakistan are subject to heightened levels of occupational stress, attributed mainly to elevated workloads, inadequate levels of support, and a demanding professional environment^53^. Similarly, a review study indicated that heightened workload is a significant factor in decreasing job satisfaction and increasing stress levels among community pharmacists, emphasizing the need for improved workload management to enhance their overall well-being and professional satisfaction^89^. Likewise, the research of Holden et al.^54^ revealed that increased cognitive demands during the dispensing process in pediatric hospital pharmacies are associated with reduced perceived medication safety and decreased employee well-being, indicating the critical importance of effective workload management in ensuring patient safety and promoting the well-being of pharmacy staff^54^. A meta-analysis across various occupational settings confirmed that workload is a significant predictor of burnout, closely linked to reduced mental well-being^90^.

The negative effects of an excessive workload on the psychological well-being of hospital pharmacists can be ascribed to a confluence of interconnected factors. Elevated workloads are associated with chronic stress and pressure, resulting in the depletion of cognitive and emotional resources and contributing to mental exhaustion^91^. The continual imposition of strain also heightens the likelihood of errors, giving rise to apprehensions regarding patient safety and professional culpability. Furthermore, it is common for pharmacists to experience inadequate intervals for recovery between strenuous tasks or shifts, which can result in prolonged stress and burnout. Excessive workloads can potentially encroach upon individuals’ time, thus disrupting the equilibrium between work and personal life and potentially resulting in social isolation and neglect of personal well-being^92^. The concurrent influence of these factors is implicated in the diminished mental health of hospital pharmacists, as evidenced in the study and substantiated by previous research. In Punjab, pharmacists often juggle dual stressors of professional responsibilities and familial obligations, which are deeply rooted in the region’s socio-cultural norms. These dual demands intensify the psychological toll of excessive workload, making effective workload management even more critical. This aligns with previous studies emphasizing the unique socio-cultural stressors healthcare professionals face in South Asia^93,94^.

### Mindfulness as a mediator leading to study hypothesis 2

In addition, the results supported the second hypothesis, positing that mindfulness plays a mediating role in this relationship. Specifically, the findings indicate that mindfulness serves as a protective factor, mitigating the negative impact of workload on mental well-being. This outcome corroborated prior scholarly work conducted by Samios^95^, Lu et al.^96^, and Cepeda-Lopez et al.^97^, which demonstrated that mindfulness served to alleviate the adverse impacts of job-related stress on the mental well-being of healthcare professionals. This notion was further supported by a systematic review of 58 articles, including randomized control trials, pre-post studies, cross-sectional, cohort, and qualitative studies, highlighting that Mindfulness-Based Stress Reduction (MBSR) was effective in enhancing mindfulness, self-compassion, and reducing levels of burnout, depression, anxiety, and stress among healthcare professionals^98^. In Pakistan, where workplace stress is widespread, implementing mindfulness practices may be especially advantageous in assisting pharmacists in coping with heavy workloads and preserving their psychological welfare. In cultural contexts like Punjab, where mental health discussions are often stigmatized^99^, mindfulness offers a personal and culturally adaptable approach to managing stress. By enhancing emotional regulation and fostering resilience, mindfulness can help pharmacists navigate workload-related stress without external reliance.

### POS as a mediator leading to study hypothesis 3

Furthermore, the study findings supported the mediating role of POS, indicating that perceptions of organizational support are crucial in mitigating the adverse effects of workload on mental well-being. This finding aligned with prior empirical evidence that underscores the importance of organizational support in the healthcare sector. For instance, Ahmad et al.^100^ found that healthcare professionals in Pakistan who perceived higher levels of organizational support reported lower stress levels and better overall mental health outcomes. Additionally, international studies corroborated these findings. Griffin and Clarke^101^ demonstrated that POS significantly mitigated the relationship between job stressors and employee strain in the United States, underscoring the universal importance of organizational support across different cultural contexts. Furthermore, research by Hsieh et al.^102^ in Taiwan highlighted that POS alleviates job burnout and enhances job satisfaction, reinforcing the critical role of a supportive work environment in promoting employee well-being. These studies collectively suggested that fostering a supportive organizational culture can significantly buffer the negative impacts of workload on mental well-being, making it a vital strategy for improving the mental health of pharmacists and healthcare workers. POS may hold heightened significance in collectivist cultures like Punjab, where workplace relationships and a sense of belonging are crucial. Pharmacists who perceive their organizations as supportive may experience a stronger psychological buffer against workload-induced stress, aligning with the region’s cultural emphasis on communal support.

### Sequential mediation of mindfulness and POS leading to study hypothesis 4

The serial mediation pathway provided evidence for our study H4, suggesting that workload can increase mental well-being through a sequential mediating process involving mindfulness and POS. This reflected a combined protective effect, where mindfulness augments perceptions of organizational support to protect mental well-being amidst challenging occupational conditions. This chain mediation effect underscored the interdependence of individual resources and organizational variables in shaping the mental well-being of pharmacists confronting significant workloads. These findings were aligned with Hulsheger et al.^71^ and Shonin et al.^72^, who demonstrated that mindfulness amplifies the perception of organizational support, creating a cascading effect on resilience and well-being. Mindfulness practices have been shown to bolster an individual’s capacity to maintain vigilance and resilience in the face of stress, leading to increased recognition of the support proffered by their organization^103,104^. This amplified perception of organizational support serves as a mitigating factor against the deleterious impact of workload on mental well-being.

### The cultural context of Pakistan

In a country like Pakistan, where resource limitations are evident^105^, just inculcating mindfulness and enhancing organizational support would likely pay dividends in mental well-being among pharmacists. Future strategies that will need to be explored within the training of pharmacists are mindfulness-based interventions, which may provide further skills training amongst pharmacists needing additional support managing their stress and workload without an increased demand on managerial time through a nurturing organizational environment. Empirical investigations indicated that such interventions can substantially improve job satisfaction and reduce burnout, enhancing overall mental well-being^71,106^. Furthermore, organizational support can be realized in many ways, including providing adequate facilities and equipment, favorable working conditions, and emotional backing from superiors. Multiple investigations have reliably demonstrated a significant correlation between higher perceived organizational support, lower stress levels, and superior mental health^67^. Building resilience and creating a psychologically healthier workforce might synergize integrating individual-focused interventions such as mindfulness with organizational strategies to increase support.

### Limitations

While the present study provides valuable insights, it has certain limitations. First, the generalizability of the findings is restricted as data were predominantly collected from hospital pharmacists in a specific geographic area. This constraint may limit the applicability of the results to pharmacists working in different contexts, healthcare systems, and workload conditions. Second, the cross-sectional design used in this study limits the ability to draw causal inferences between workload and mental well-being, as it captures only a single point in time, precluding an understanding of temporal relationships and causality. Third, relying on self-reported measures introduces the risk of response bias, where participants’ accounts of their workload and mental well-being might be affected by social desirability or recall inaccuracies. Fourth, while the study conceptualized POS as a mediating variable based on established theoretical frameworks and empirical evidence, it is also possible to conceptualize POS as a moderator. This perspective suggests that POS may buffer the relationship between workload and mental well-being by mitigating the adverse effects of job stressors. Future research could explore this possibility within a multilevel framework, particularly by examining POS as an organizational climate variable. Alternatively, Perceived Supervisor Support (PSS) could be investigated as an individual-level mediator to understand the mechanisms of support further. Lastly, despite controlling for several confounding variables, the potential influence of unmeasured confounders, such as personal coping mechanisms or organizational culture, cannot be entirely ruled out, potentially affecting the observed relationships.

## Conclusion

The present research provides compelling evidence of the challenges posed by excessive workload on the mental well-being of hospital pharmacists. By exploring direct and mediated pathways, this study offers practical insights for improving workplace environments and supporting healthcare workers. Key conclusions are summarized as follows:

- This study confirms the direct negative impact of workload on the mental well-being of hospital pharmacists, a challenge that resonates across various healthcare settings.
- The findings highlight the importance of interventions within workplace or practice settings to address workload-related problems and improve pharmacists’ mental health.
- Policy changes are recommended to monitor and mitigate the adverse effects of excessive workload on healthcare workers.
- Promoting better mental health among pharmacists is crucial for their welfare and enhancing patient safety and care.
- While this research provides essential insights, future studies should address its limitations to deepen our understanding of workload, mental well-being, and their mediating mechanisms.

## Data Availability

The raw data that support the findings of this study are available upon reasonable request from the first author (Shazia Rehman, rehmanshaiza.malik@gmail.com).
